# Protection induced by a *Francisella tularensis* subunit vaccine delivered by glucan particles

**DOI:** 10.1371/journal.pone.0200213

**Published:** 2018-10-08

**Authors:** Adam O. Whelan, Helen C. Flick-Smith, Jane Homan, Zu T. Shen, Zoe Carpenter, Payam Khoshkenar, Ambily Abraham, Nicola J. Walker, Stuart M. Levitz, Gary R. Ostroff, Petra C. F. Oyston

**Affiliations:** 1 CBR Division, Dstl Porton Down, Salisbury, United Kingdom; 2 ioGenetics LLC, Madison, WI, United States of America; 3 University of Massachusetts Medical School, Worcester, Massachusetts, United States of America; New York Medical College, UNITED STATES

## Abstract

*Francisella tularensis* is an intracellular pathogen causing the disease tularemia, and an organism of concern to biodefence. There is no licensed vaccine available. Subunit approaches have failed to induce protection, which requires both humoral and cellular immune memory responses, and have been hampered by a lack of understanding as to which antigens are immunoprotective. We undertook a preliminary *in silico* analysis to identify candidate protein antigens. These antigens were then recombinantly expressed and encapsulated into glucan particles (GPs), purified *Saccharomyces cerevisiae* cell walls composed primarily of β-1,3-glucans. Immunological profiling in the mouse was used to down-selection to seven lead antigens: FTT1043 (Mip), IglC, FTT0814, FTT0438, FTT0071 (GltA), FTT0289, FTT0890 (PilA) prior to transitioning their evaluation to a Fischer 344 rat model for efficacy evaluation. F344 rats were vaccinated with the GP protein antigens co-delivered with GP-loaded with *Francisella* LPS. Measurement of cell mediated immune responses and computational epitope analysis allowed down-selection to three promising candidates: FTT0438, FTT1043 and FTT0814. Of these, a GP vaccine delivering *Francisella* LPS and the FTT0814 protein was able to induce protection in rats against an aerosol challenge of *F*. *tularensis* SchuS4, and reduced organ colonisation and clinical signs below that which immunisation with a GP-LPS alone vaccine provided. This is the first report of a protein supplementing protection induced by LPS in a *Francisella* vaccine. This paves the way for developing an effective, safe subunit vaccine for the prevention of inhalational tularemia, and validates the GP platform for vaccine delivery where complex immune responses are required for prevention of infections by intracellular pathogens.

## Introduction

*Francisella tularensis* is an intracellular pathogen and the causative agent of the disease tularemia. Capable of infecting a wide range of hosts, its normal zoonotic hosts are rodents and lagomorphs, but humans can be accidental hosts. In humans, the most acute presentation is respiratory or pneumonic tularemia, following the inhalation of infectious aerosols: by this route the organism has a very low infectious dose for humans, requiring under 50 CFU to establish respiratory infection [1]. Following inhalation, the most highly virulent strains can have a case fatality rate of up to 30% if untreated, but appropriate antibiotic therapy reduces this to around 2% [2]. Diagnosis based on symptoms is difficult as the presentation can range from a mild pneumonia to an acute infection with high fever, malaise, chills, cough, delirium and pulse-temperature dissociation, all of which are very non-specific. The high aerosol infectivity, morbidity and mortality, led to the organism being developed as a biological weapon by various nations, including the reported production of antibiotic resistant strains [2,3]. As such, there is strong interest in developing effective medical countermeasures to prevent and treat tularemia.

No licensed vaccine is available for prevention of tularemia. Killed whole cells, subunits and live attenuated strains have all been evaluated historically. Killed whole cell preparations were reactogenic and of dubious efficacy [4,5], although studies in humans indicated that immunization with these vaccines reduced the number of infections and severity of disease. A live vaccine strain (LVS) was developed in the 1950s, and significantly decreased laboratory acquired infections in at-risk workers [6]. In human volunteer trials, LVS induced protection against an aerosol challenge with 10 infectious doses of a virulent strain, but induced poorer protection against higher challenge levels [7]. There have been concerns about the LVS strain, such as reversion to virulence, mixed colony morphology and variable immunogenicity, and thus to date the LVS strain has failed to achieve licensing for human use [8]. The development of a defined attenuated strain more acceptable for licensing continues to be the focus of much research, based on the previous success of the LVS strain in inducing protection. The identification of the antigens for a subunit vaccine has similarly been explored for many years. To date the only protective antigen identified is lipopolysaccharide (LPS). In humans the predominant antibody response is to LPS. However, while immunisation of animals with LPS induced protection against *F*. *tularensis* strains of low virulence, it was less effective at providing protection against highly virulent strains [9–12]. To date, many immunogenic proteins have been identified [13], but none capable of inducing a protective immune response. This is probably a reflection that antibody is not sufficient to protect against tularemia and a T cell memory response must be induced for a vaccine to be protective [14]. It may also be a reflection of the highly acute nature of the murine tularemia model, which most vaccine screens have been undertaken in. In the early days of tularemia research, the limitations of the available animal models meant that researchers felt that human infection was the only valid way to study the disease and develop countermeasures (reviewed by [15]). Subsequently primate, mouse, rat, rabbit, and guinea pig models were developed, all of which had different strengths and weaknesses when extrapolating to the human (reviewed by [15]). Nowadays, the majority of *in vivo* tularemia studies are undertaken in the mouse as it is relatively low cost and there are many tools available for establishing host responses. However, the mouse is significantly more sensitive to *F*. *tularensis* than humans and responses to vaccination also differ: protection induced by LVS is not similar to that observed in humans, particularly to respiratory challenge, and the protection is short-lived. Due to the increased size of animal and higher associated costs, the rat fell out of favour for some time. However, once the deficiencies of the mouse model as a vaccine protection model became clear it received renewed attention [16–18]. Rats are more resistant to *F*. *tularensis* than mice, and LVS protects rats similarly to humans. Therefore, although mice have provided valuable insights into vaccine immunity, and remain an important tool model for investigating immunogenicity, rats are increasingly viewed as being potentially more appropriate for efficacy studies.

That killed cell preparations have been shown to induce partial protection in humans against tularemia implies that a subunit vaccine should be achievable, if protective subunits can be identified and delivered in a way that stimulates the correct immune response. Many immunogenic *Francisella* subunits have been proposed as potential vaccine candidates [19–23]. In our own laboratory, various approaches have been undertaken to identify suitable antigens, including screening for polypeptides able to induce the proliferation of T-cells taken from humans immunized with the LVS vaccine or who had recovered from tularemia. We undertook a screen using a protein microarray probed with sera from mice immunized by intramuscular injection with killed LVS adjuvanted with preformed immune stimulating complexes (ISCOMs) admixed with CpG: these mice were protected when systemically challenged with the highly virulent strain of *F*. *tularensis* SchuS4 [24]. The five proteins which had induced the highest titres in these mice were adjuvanted with Provax^TM^, a squalene/Tween 80 based adjuvant capable of inducing MHC Class I-restricted cytotoxic T cell responses [25], and used to immunize mice. When these mice were challenged with *F*. *tularensis*, none survived. Subsequently, proteins recognized by sera from immunized or convalescent individuals exposed to subspecies *tularensis* were also evaluated. Sixteen recombinant *Francisella* proteins adjuvanted with ISCOMS and CpGs were used to immunize mice; again no protection was observed against challenge with strain HN63 (unpublished data). Finally, we made the antigen selection based on T cell responses by spleen cells taken from LVS immunized mice. These antigens were adjuvanted with ISCOMS and CpGs and used to immunize mice, but failed to protect against challenge with SchuS4 (unpublished data). An alternative approach to the identification of protective sub-units that we have undertaken was an analysis of amino acid composition to identify composition associated with effective vaccine antigens [26], which identified further potential vaccine candidates. However, when any of the proteins identified by all these diverse approaches were evaluated as protective sub-unit vaccines in the murine model of disease with a range of adjuvants, they were immunogenic, but did not induce protection against disease (unpublished data).

We hypothesised that we need to deliver the promising candidate antigens in a manner that induces both humoral and cellular immune responses to achieve protection. In this paper, we describe the use of glucan particle (GP) technology for antigen delivery [27]. β-glucan particles (GPs) are purified *Saccharomyces cerevisiae* cell walls, treated so that they are primarily β1,3-D-glucans and free of mannans, proteins and nucleic acids. GPs are phagocytosed by dendritic cells (DCs) via the dectin-1 receptor, so that associated antigens are processed and presented to stimulate cell-mediated immunity (CMI) in a milieu of proinflammatory cytokines secreted by the DCs [28]. GPs have been exploited as a receptor-targeted vaccine delivery system to induce both cellular and humoral immune responses [29]. The hollow, porous GP structure allows for high antigen loading of single or multiple antigens and the co-delivery of antigen(s) together with co-encapsulated adjuvants to tailor the desired immune response. The efficiency of targeted antigen/adjuvant co-delivery results in strong immune responses at reduced dosage levels (24). In this study, we loaded GPs with purified antigens (both proteins and LPS) from *F*. *tularensis* and immunized mice. Immune responses in inbred mice and computational analysis facilitated down-selection of the most promising candidates. These were then evaluated in inbred Fischer F344 rat to assess their ability to induce protective immune responses.

## Materials and methods

### Strains and culture conditions

*F*. *tularensis* LVS was derived from an original NDBR 101, Lot 4 vaccine ampoule produced during the 1960s, which had been stored at ^-^20°C. Bacteria were reconstituted according to manufacturer’s instructions, cultured overnight at 37°C on supplemented blood cysteine glucose agar (BCGA) and harvested into sterile phosphate buffered saline (PBS, pH 7.2) and stored at ^-^80°C as single use aliquots. *F*. *tularensis* SchuS4 was cultured at 37°C on BCGA, or modified cysteine partial hydrolysate (MCPH) broth. For preparation of cultures for aerosol infection studies, *F*. *tularensis* SchuS4 was first grown for 24 h on BCGA and the harvested bacteria then used to inoculate MCPH broth which was incubated for a further 48 h at 37°C, shaking at 180 rpm. To allow bacterial enumeration, cultures were serially diluted in PBS and plated on BCGA. BCGA plates were supplemented with lincomycin, colistin sulphate, amphotericin B and trimethoprim (LCAT) selective supplement (Thermo Scientific) to aid bacterial enumeration from animal tissues. *Escherichia coli* were cultured on Luria Bertani (LB) plates or broth. Unless stated all chemicals were purchased from Sigma-Aldrich.

### Protein purification

The proteins evaluated as potential vaccines in this study are listed in Table 1. IglC clone FtCD00062598 was sourced from the DNASU plasmid repository [30], but the majority of expression plasmids were produced in-house. The majority of the proteins were expressed recombinantly as His_6_-tagged proteins, with the exception of FTT1078, FTT0814, and FTT0890, which were expressed as glutathione-S-transferase (GST) fusions in *E*. *coli* BL21, with GST tags subsequently cleaved. *E*. *coli* BL21 DE3 pLysS (Invitrogen) harbouring recombinant pGEX-4-T3 or pCRT7/NT-TOPO plasmids were cultured in LB-broth containing 50 μg/ml ampicillin, 30 μg/ml chloramphenicol and 1%w/v glucose. Cultures were grown with shaking (180 rev min^-1^) at 37°C to an A_600nm_ of 1.0 prior to induction with 1.0 mM IPTG. Cultures were incubated for a further 4 h, followed by harvesting by centrifugation at 18 600 g for 15 mins.

**Table 1:** *F*. *tularensis* antigens tested.

	Putative Protein Function
FTT0071	Citrate synthase, GltA
FTT0143	L7/L12 50S ribosomal protein
FTT0209	Homology with pneumococcal surface antigen A, PsaA
FTT0239	MurC / UDP-N-acetyl-muramate:alanine ligase
FTT0289	Putative lipoprotein of unknown function
FTT0374	CTP synthase
FTT0438	UDP-N-acetylmuramate:L-alanyl-gamma-D-glutamyl-meso-diaminopimelate ligase /murein peptide ligase
FTT0464	L-asparaginase II ansB / periplasmic L-asparaginase II precursor
FTT0468	Peptidyl-prolyl cis-trans isomerase
FTT0482	Unknown
FTT0540	Unknown
FTT0547	Unknown
FTT0721	Catalase peroxidase, KatG^[^^22^^]^
FTT0724	Penicillin binding protein (D-alanyl-D-alanine carboxypeptidase)
FTT0814	Unknown
FTT0890	Type IV pilin protein, PilA
FTT0901	Outer membrane 17 kDa lipoprotein LpnA or Tul4-A ^[^^23^^,^^42^^–^^44^^]^
FTT0904	Outer membrane protein Tul4-B ^[^^43^^,^^44^^]^
FTT0918	Outer membrane protein YapH-N ^[^^43^^,^^44^^]^
FTT1043	Macrophage infectivity, Mip ^[^^43^^,^^44^^]^
FTT1161	Adenylate kinase
FTT1357/FTT1712	23 kDa protein, IglC (two loci present in SchuS4)^[^^22^^,^^45^^]^
FTT1416	Putative lipoprotein of unknown function
FTT1419	Unknown
FTT1425	NADH oxidase
FTT1696	Heat shock protein 60, Hsp60 ^[^^24^^]^
FTT1754	Phosphate acetyltransferase
FTT1768	Endochitinase/chitinase family protein

For initial purification of His-tagged proteins, cell pellets were resuspended in Bugbuster® (MerckMillipore) (5 mL per g of cell pellet), incubated for 20 min at room temperature followed by centrifugation 20,000 g for 1 h. Soluble proteins were added to Ni Sepharose 6 Fast Flow (GE Healthcare) equilibrated with Bugbuster® and incubated rolling for 2 h. The resin was then packed into a column and washed with 20 mM sodium phosphate, 500 mM NaCl, 10% glycerol pH7.7 containing 50 mM imidazole. Bound protein was eluted with 200 mM imidazole in the same buffer. For insoluble proteins following treatment with Bugbuster® pellets were washed with 1% Triton-X100 in PBS, followed by 1 M NaCl prior to resuspension in 8 M urea in PBS. Following incubation over night the suspension was centrifuged 20,000 g for 1 h and the supernatants purified as above but with all buffers containing 8 M urea. Subsequently, His-tagged proteins were purified as described above, but with an additional wash with 0.1% triton-X114 in PBS whilst bound to the Ni Sepharose 6 Fast Flow (GE Healthcare) to remove endotoxin.

For purification of GST tagged proteins, cell pellets were resuspended in PBS (5 mL per g of cell pellet), and lysed by sonication followed by centrifugation 27,000g for 30 min. The supernatant was loaded onto a GSTrap HP column (GE Healthcare) equilibrated with PBS and washed to baseline with PBS. Thrombin protease (GE Healthcare) 80 units per mL bed volume in PBS was loaded onto the column and incubated overnight. Cleaved protein was washed from the column with PBS.

Affinity purified soluble proteins were dialysed into 20mM Tris pH7.5 and insoluble ones into the same buffer containing 8M urea prior to loading onto a 1 mL CaptoQ column (GE Healthcare) equilibrated with the respective buffers. Columns were washed with the same buffer plus 0.01 mM NaCl prior to elution with 0.01 to 1 M NaCl gradient in the same buffer. Purified insoluble proteins were refolded following concentration to 3 to 5 mg/mL by dilution in 10 volumes of refolding buffer (PBS, 400 mM NaCl, 20% glycerol, 0.5 mM EDTA, 0.1% Tween) and then dialysed into PBS plus 0.5 M urea.

As required, endotoxin levels were depleted by passing through Detoxi-gel™ endotoxin removal columns (Thermo Scientific). The protein concentration was assessed by Bicinchoninic Acid (BCA) assay (Thermo Scientific), and purity assessed by SDS-PAGE and densitometry following Coomassie staining.

### Polysaccharide purification

The LPS of *F*. *tularensis* subspecies *tularensis* and subspecies *holarctica* have been shown to be identical. Therefore, for initial GP formulation and optimisation experiments, LPS was extracted from the LVS strain. Briefly, LPS was extracted from freeze-dried bacteria with 45% phenol at 67°C. The resulting pellet was re-extracted and the water phases from the two extractions were dialysed against water for 3 days. The resulting solution was ultracentrifuged and treated with RNase and proteinase K. SchuS4 derived LPS was generously provided as a gift by W. Conlan, Health Canada. The SchuS4 derived LPS was used in all GP animal vaccination experiments.

### Glucan particles

GPs were prepared from *S*. *cerevisiae* (Fleischmann’s baker’s yeast) using a series of alkaline and acidic extraction steps as previously described [31,32]. Briefly, following centrifugation and washing in water, *S*. *cerevisiae* was subjected to two rounds of hot alkali extraction by heating for 1 h at 90°C in 1 M NaOH. The particles were suspended in water at pH 4.5, heated at 75°C for 1 h, and then successively washed with water (three times), isopropanol (four times), and acetone (two times). GPs were dried to yield a free-flowing light tan powder. To count the GPs for GP vaccine formulations, a 10 μg/ml suspension of particles in 0.9% saline was lightly sonicated, counted using a hemocytometer, and then kept in aliquots at ^-^20°C until use. One microgram of GPs contains approximately 5x10^5^ particles.

### GP vaccine formulations

GPs containing encapsulated core-loaded *Francisella* antigen complexed with ovalbumin (OVA) and Torula yeast RNA were prepared as described previously (73). Briefly, 10 mg of dry GPs were swollen with 50 μl of 10 mg/ml *Francisella* antigen dissolved in water or 6 M urea at ambient temperature to minimally hydrate the GPs, allowing the soluble antigen to diffuse into the hollow GP cavity. The samples were then frozen at ^-^80°C and lyophilized. After lyophilization, the same procedure was repeated to load the selected albumin into the particles. To maximize *Francisella* antigen and albumin encapsulation into the GP shells, the dry GP *Francisella* antigen-albumin formulations were swollen, mixed with 25 μl of sterile water and lyophilized. To trap the antigen and albumin inside the GPs, the dry GP antigen-albumin formulations were heated to 50°C and swollen with 50 μl of 25 mg/ml yRNA (derived from torula yeast, type VI) in 0.15 M NaCl for 30 min, and then 10000 μl of 10 mg/ml yRNA was added for 1 h at 50°C to complete the complexation reaction, trapping both the *Francisella* antigen and albumin inside the GPs. The suspension was centrifuged and washed three times in 0.9% saline, and particles were resuspended in 70% ethanol, incubated 30 min at room temperature to sterilize, aseptically washed three additional times in sterile 0.9% saline, resuspended, counted, diluted to 1x10^9^ particles/ml in sterile 0.9% saline, and stored at -80°C. To calculate the amount of protein encapsulated into the GPs, rhodamine-labelled bovine serum albumin (BSA) or OVA was prepared by reaction with rhodamine B isothiocyanate (RITC) and used as a fluorescent tracer to estimate albumin incorporation into the GP particles as compared to the unbound in the saline washes. Typically, albumin encapsulation efficiency was >95%.

To produce GPs with LPS on the particle surface, an avidin bridge was used. Briefly, 5x10^9^ biotinylated GPs were incubated with 500 μl of 1 mg/ml avidin at 4°C for 1 hour. The avidin-GPs were extensively washed in 0.9% saline to ensure that the unbound avidin was removed. *F*. *tularensis* LPS was biotinylated with biotin hydrazide using an excess of biotin hydrazide to LPS ranging from one- to five-fold. Next, the avidin-GPs were incubated with 100 μl of 5 mg/ml biotinylated *Francisella* LPS for one hour at 4°C. GPs were extensively washed in 0.9% saline to ensure that the unbound biotinylated *Francisella* LPS was removed.

To confirm encapsulation efficiency and demonstrate antigen identity inside of the loaded GPs, 10% SDS-PAGE analysis was undertaken. For each GP vaccine, input antigen, GPs containing core loaded antigens and OVA and supernatants from the first wash after completing the loading reaction were evaluated to provide a test for successful antigen loading and antigen identity. Sterility of the GP vaccines was confirmed by culture of an aliquot.

GPs co-loaded with antigen and rhodamine-labelled OVA were imaged using a high-resolution Zeiss (Thornwood, NY) Axiovert 200 inverted microscope equipped with a Zeiss AxioCam HR CCD camera with 1,300 × 1,030 pixels basic resolution and a Zeiss 100 × 1.40 NA oil-immersion objective. Differential interference contrast (DIC) and fluorescent micrographs of the same section of GPs were generated for proper comparison.

For vaccinations studies, each 0.1ml vaccine dose contained 10μg of antigen (protein and/or LPS) encapsulated in 200μg of GP particles (1x10^8^ GP particles).

### Ethics statement

Animals were kept, and procedures performed, in accordance with the UK Animals (Scientific Procedures) Act 1986. The licence application underwent approval by the local ethical review process with the Dstl Animal Welfare and Ethical Review Body (AWERB) before submission and approval with the UK Home Office and Animal Procedures Committee (an independent committee that offers advice to The Secretary of State of the ethics of the proposed work). The project licence that covered this work was 30/3166. The animal studies were also performed under the authority of an application approved by the US Animal Care and Use Review Office (ACURO).

### Animal procedures

Animals were randomly allocated into cages upon arrival and housed under a 12-h light/dark cycle with free access to food and water. No animals were excluded from the study. Female 6- to 8-week old Balb/c and C57BL/6 mice were supplied by Charles River Laboratories, UK. For intramuscular (im) dosing, mice were immunized with 50 μl in each hind limb (100 μl per dose). Subcutaneous (sc) and intraperitoneal (ip) dosing typically used 100 μl volumes containing 10μg of each antigen and 200μg of GP particles. Mice received three immunisations 2 weeks apart. Six weeks following the final vaccination, mice were either culled by cervical dislocation for immunological screening or challenged with *F*. *tularensis* SchuS4 via an ip delivery route (100 μl). Mouse welfare checks were performed a minimum of twice daily following challenge, and clinical signs were recorded for each mouse. Clinical signs recorded included presence of piloerection, hunched posture, reduced mobility, abnormal breathing rate and problems associated with one or both eyes. Animals that were moribund and deemed incapable of recovery were culled according to pre-determined humane end-point criteria. Cull criteria were lack of mobility, paralysis of any limb, or problems with both eyes.

Female 6- to 8-week old Fischer 344 (F344) rats were supplied by Envigo, UK. F344 rats were immunized sc with GP vaccines in a 100 μl volume using the same vaccination schedule as used for mice. In addition, control groups of rats were immunized with a single 100 μl sc injection of 1x10^7^ cfu LVS. A tail bleed was performed 2-weeks after the final GP-vaccination, or 4 weeks after the LVS vaccination, for immunology. Six weeks following the final vaccination, rats either underwent euthanasia by ip administration of sodium pentobarbitone for immunological assessment or they were challenged with *F*. *tularensis* SchuS4 via an aerosol delivery route. The aerosol was delivered by inhalation in a nose-only exposure unit utilising a 3-jet Collison atomiser attached to a contained Henderson Piccolo arrangement to condition the aerosol to 50% (±5%) relative humidity, and controlled by the (AeroMP) Aerosol Management Platform aerosol system (Biaera Technologies L.L.C.). Groups of 5 rats were exposed to the aerosolised bacteria for 10 min, with impingement of the aerosol cloud sampled at the midway point of challenge into PBS via an All-Glass Impinger. (AGI-30; Ace Glass, Vineland, NJ). Following challenge, rats were checked at least twice daily, and clinical signs, as reported for mice, recorded. Individual rat weights were recorded daily. Rats were monitored for 14 days post-infection and culled when they reached pre-determined humane end-point criteria. The cull criteria for rats was >15% weight loss and/or moribund and deemed incapable of recovery, as described for infected mice. All rats that survived to the end of the study were euthanized to allow bacterial enumeration in lung and spleens.

There were no unexpected deaths of any study mice or rats. Analgesia was not administered to mice or rats displaying clinical signs due to the potential confounding influence on disease progression. Animal suffering was minimised by increasing welfare checks to a minimum interval of 8 hours during the acute phase of the infection to ensure timely application of humane endpoints where required.

### IVIS imaging study

To evaluate the effect of administration route on subsequent dispersal of the GP vaccines, Balb/c mice in groups of 3 were immunized by either the sc or im route with either Cy7 labelled GPs (Cy7-GP-OVA alone or with Ft LPS (Cy7-GP-OVA+LPS or GPs containing Cy7 labelled OVA (GP-Cy7-OVA) alone or with Ft LPS (GP-Cy7-OVA+LPS). The GPs were administered on day 1 and mice were imaged daily for 5 days. Prior to imaging, fur was removed from mice by shaving. For imaging, mice were anaesthetised using 0.78 mg ketamine-medetomidine (Ketaset, Fort Dodge Animal Health Ltd, UK) and 0.015mg Domitor (Elanco, UK) given in a total volume of 150 μl ip. Once unconscious, mice were imaged using an IVIS Spectrum (Caliper, Perkin Elmer, USA) and images captured and analysed using the Living Image 4.5 software. Fluorescent signal was detected at excitation 710 nm and emission 760 nm. After imaging, mice were recovered using 0.05 mg in 100 μl ip of Atipamezole (Antisedan, Janssen Animal Health, UK). Whilst under anaesthetic mice were kept in a warming box and closely observed until fully recovered. After imaging on day 5 mice were culled by cervical dislocation and lung, liver, spleen and selected lymph nodes (popliteals and inguinals) were also imaged separately.

### TNFα bioassay

Bone marrow derived dendritic cells (BMDCs) were generated as previously described with a slight modification [33,34]. Briefly, bone marrow cells obtained from the tibiae and femurs of 8- to 12-week-old C57BL/6J mice (Jackson Laboratories) were cultured in R10 medium supplemented with 10% GM-CSF conditioned medium from the mouse GM-CSF–secreting J558L cell line. Cells were fed with fresh GM-CSF–supplemented R10 on days 3 and 6. On day 8 adherent cells were collected and plated in 96-well plates, at a density of 10^5^ cells/ml (100 μl) in 96 well plates using GM-CSF-supplemented R10 medium. Samples (10 μl) were added to BMDCs and incubated overnight at 37°C, 5% CO_2_ to stimulate TNFα secretion. PBS and empty GPs (5 particles/cell) served as negative control, *E*. *coli* LPS (100 ng/ml) served as positive control. The supernatant was assayed for TNFα using an ELISA kit for TNFα (Ebioscience) following manufacturer’s instructions.

### Isolation and culture of lymphocytes

Splenocytes were isolated by maceration of rat or mouse spleens through a 40μm cell sieve. For mouse splenocyte isolation, red blood cells (RBCs) were lysed by incubation of cells for 5 min in Red Blood Lysis buffer (Sigma-Adrich) following by 2 washes with PBS. Rat splenocytes were isolated directly from macerated spleens without the use of a RBC lysis step. For isolation of rat PBMCs, blood was collected from a tail vein (200–1000μl) into heparin containing collection tubes (Sarstedt), diluted into 2ml of PBS and overlayed onto 2ml of Ficoll-Paque 1.084 gradient density centrifugation media (GE Healthcare, UK). PBMCs from individual rats were isolated by centrifugation at 800 g for 40 min followed by 2 washes with PBS. Following lymphocyte enumeration, cells were diluted in RPMI1640 medium (Life Sciences, UK) supplemented with 10% Foetal Bovine Serum (Sigma), non-essential amino acids (Life Technologies), 2-mercaptoethanol (Life Technologies), 100U/ml penicillin and 100mg/ml streptomycin sulphate (Life Technologies). Murine splenocytes (1–5 x 10^5^ cells) were added to duplicate wells of either flat bottomed 96-well microtiter plates, or to pre-coated murine IFNγ ELISPOT plates (BD Biosciences, UK). Rat splenocytes (2.5x10^5^ cells) were added to duplicate wells of flat bottomed 96-well microtiter plates. Rat and mouse PBMC (1x10^5^ cells) were added to duplicate wells of round bottomed 96-well microtiter plates. Splenocytes or PBMC cultures were then incubated at 37°C/5% CO_2_ in the presence of medium alone, individual protein antigens (5 μg/ml, Dstl), LVS lysate (10 μg/ml, Dstl) or Con-A (5 μg/ml, Sigma-Aldrich). For the mouse and rat PBMC cultures only, cells were co-stimulated with 5μg/ml of anti-mouse CD28 (clone 37.51, BD Biosciences) or anti-rat CD28 (clone JJ319, eBioscience) antibodies respectively. To allow measurement of murine IFNγ ELISPOT responses, cultures were incubated for 16–20 h. To allow measurement of cytokines by IFNγ ELISA or CBA assay, cultures were incubated for 72 h.

### J774A.1 macrophage/splenocyte co-culture killing assay

The macrophage/splenocyte co-culture killing assay was a modification of a method from Roberts *et al* [35]. In summary, murine splenocytes were isolated as described above and cultured in L15 medium (Life Sciences, UK) with either medium alone, 10 ng/ml phorbol myristate acetate(PMA; Sigma) + 1ug/ml ionomycin (Sigma) or heat killed *F*. *tularensis* SchuS4 for 20 h at 37°C. The murine macrophage cell line J774A.1 (Public Health England, ECACC, 91051511) was propagated in antibiotic-free RPMI1640 medium supplemented with 10% FBS and seeded in 48-well plates at 5x10^5^ cells/well. *F*. *tularensis* SchuS4 was added to J774A.1 cells at a multiplicity of infection of 10. After 90 min, the medium was removed and L15 containing 50 μg/ml gentamicin (Life Technologies, UK) was added for 45 min. The cells were washed prior to addition of the antigen-stimulated splenocytes from vaccine-immunized mice. The infected J774A.1 cells and antigen-stimulated splenocytes were then cultured in triplicate for 48 h at 37°C. Cells were then lysed in water and intracellular bacteria enumerated by plating serial dilutions in triplicate on BCGA plates.

### Cytokine assays

Mouse IFNγ ELISPOT responses were measured in 16–20 h antigen-stimulated splenocyte cultures using a commercial detection kit (BD Biosciences). The assay was performed in accordance with kit instructions and spot enumeration was performed using an AID automated reader. IFNγ in 72 h antigen-stimulate culture supernatants was detected using commercial (Mabtech) mouse and rat IFNγ ELISAs. The respective ELISA assays were performed in accordance with kit instructions and responses determined by measurement of optical density at 450nm (OD_450nm_) using a Multiskan Ascent plate reader (ThermoFisher Scientific). IFNγ concentrations were calculated from a standard curve generated using the IFNγ standards supplied with the respective kits. Antigen stimulated mouse splenocyte cultures were analysed for IL6, IL10, MCP1, IFNγ, TNF and IL-12 using a Cytometric Bead Array Assay (CBA). The CBA mouse inflammation assay kit (BD Biosciences) was performed in accordance with kit instructions and samples analysed on a FACS Canto Flow cytometer (BD Biosciences).

### IgG antibody assays

Antigen specific IgG responses were measured in serum from mice, or plasma from rats. In addition to measurement of total IgG, mouse serum samples were also assayed for antibody isotypes IgG1 and IgG2a or IgG2c depending on mouse strain. High protein binding 96-well microtitre plates were coated overnight with 5 μg/ml of antigen. In addition, selected wells were coated with goat anti-mouse antibody binding fragment (M4155, Sigma-Aldrich) or goat anti-rat IgG (R5130, Sigma-Aldrich) for standard curve calculation. Serial dilutions of mouse serum, rat plasma, or respective mouse or rat IgG standard, were added to the respective wells of coated plates and incubated overnight. Bound antibody was detected using a sequential combination of horse radish peroxidase conjugated goat anti-mouse IgG (10355), or isotypes (STAR123P and STAR133P, BioRad) or goat anti-rat IgG (A9037, Sigma) followed by 3,3′,5,5′-Tetramethylbenzidine (Sigma-Aldrich) development substrate. Responses were detected by measurement of OD_450nm_ on a Multiscan Ascent plate reader and antibody concentrations calculated using the Ascent software.

### Computational analysis

A computational platform previously described [36,37], was used to evaluate the seven lead proteins. This platform integrates predictions of multiple components of the immune system including predictions of protein topology and epitope exposure, predicted linear B cell epitopes, affinity of MHC I and MHC II binding, probability of cleavage by cathepsin, and the frequency of occurrence of the amino acid motifs which engage T cell receptors (T cell exposed motifs or TCEM) when peptides are bound in either MHC I or MHC II molecules [38]. The components of the analysis are shown in Supplemental S1 Table. An analysis of the predicted B and T cell epitopes and relative dominance of these was generated for Balb/c and C57BL/6 mice and for 35 MHC I and 28 MHC II human alleles. In addition, frequency patterns of TCEM were analysed to identify those which occur with high frequency, relative to self-protein and immunoglobulin reference databases, and thus potentially attract a large pre-existing cognate T cell population which may indicate immunosuppression [38]. An estimation of the conservation of each protein was made by comparing the presence of one or more members of the same protein family [39] in 100 isolates of *F*.*tularensis* downloaded from PATRIC on 30 November 2016 [40]; exact sequence conservation was not assessed.

### Statistical analysis

All statistical analysis of animal study data was performed in Graphpad Prism software. All data sets were tested for normality and appropriate statistical tests applied. Statistical tests, where performed, are stated in the appropriate results section and/or in the supporting figure legend. For the analysis of bacterial burdens in the tissues from rats, data was log transformed and a value of one was added to all values to allow comparative analysis of data where no bacteria were detected.

## Results

### Production of vaccines

A panel of proteins (Table 1) was selected for evaluation in the GP platform. The majority of the antigens were identified by the approach described in [26]. Previous work where the antigen has been evaluated as a potential vaccine antigen is cited in Table 1. These proteins were expressed recombinantly in *E*. *coli* and purified. Initial expression analysis showed some of the proteins to be relatively insoluble. As a result, our standard GP loading conditions were modified to include 6 M urea, which was subsequently removed by washing away the soluble urea from the GP encapsulated antigen-serum albumin-yeast RNA trapped complexes with saline [41]. Efficient GP particle loading with proteins was confirmed using three different methods. Firstly, incorporation of a tracer rhodamine-labelled OVA was monitored to estimate GP antigen encapsulation efficiently: routinely greater than 95% incorporation of the fluorescent albumin protein was observed (shown for the representative antigen FTT0814 in S1A Fig). Secondly, differential interference contrast (DIC) and fluorescent microscopy was used to qualitatively demonstrate antigen-encapsulation inside the hollow GP cavity. Finally, SDS-PAGE was used to confirm *F*. *tularensis* antigen identity, loading efficiency and integrity after GP loading (S1B Fig).

**Fig 1:**
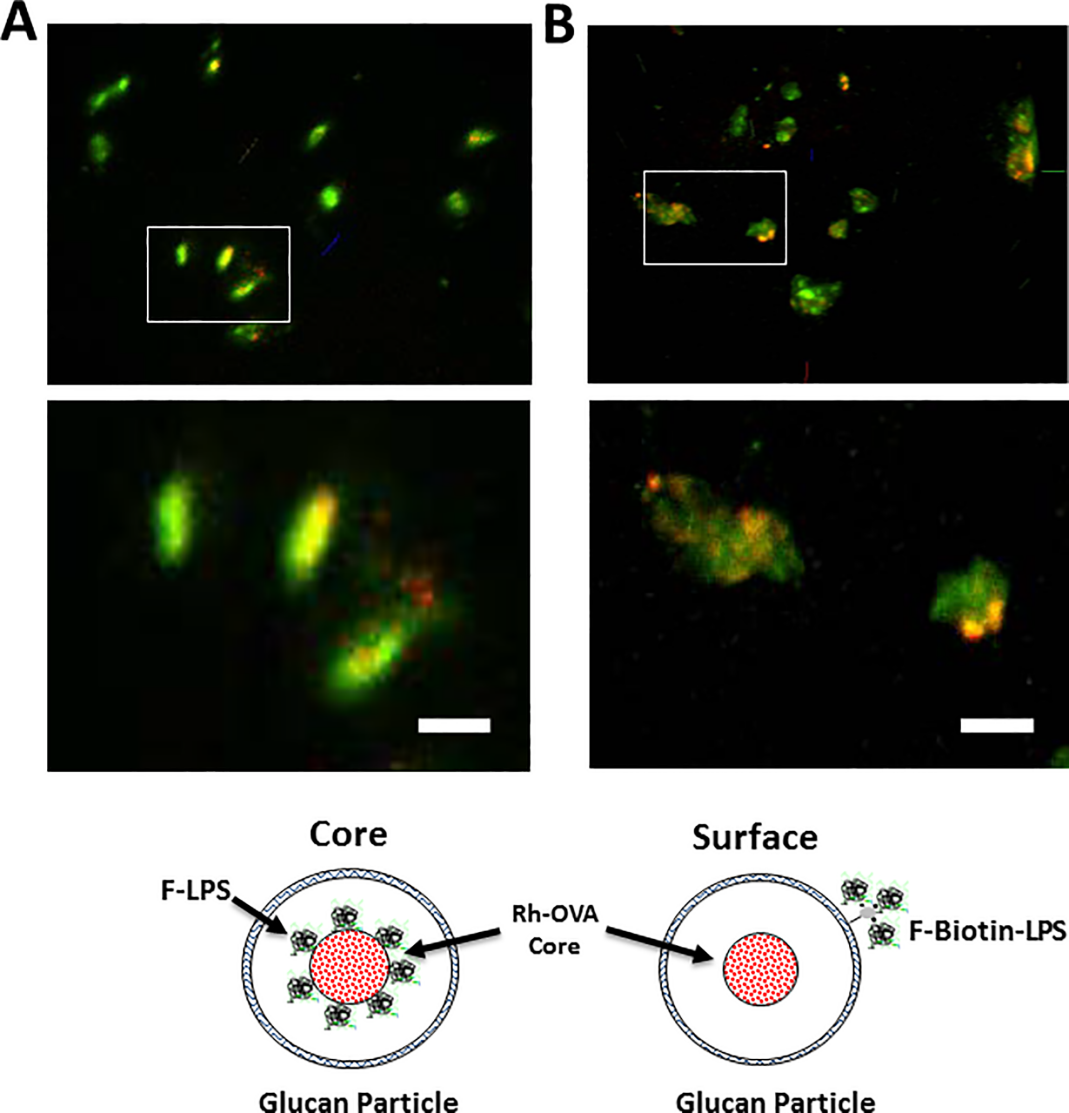
Confocal microscopy distinguishes between GPs containing core loaded or surface linked LPS. Panel A shows images of GPs core co-loaded with both Dylight 633-labelled OVA (red) or DTAF-labelled *Francisella* LPS (green) antigens. Panel B shows biotinylated GPs core loaded with Dylight 633 OVA and then surface bound to biotinylated DTAF-LPS via a streptavidin linker as also shown graphically with the cartoon. The punctate yeast shell bound F-LPS appears slightly larger than the internally loaded LPS-OVA cores. Size marker on magnified lower images = 1 μm.

The loading of GPs with *Francisella* LPS was evaluated by two methods, either core loading inside the GPs or on the GP surface via a streptavidin-biotin linkage. The efficiency of the respective loading procedures was assessed quantitatively by indirectly measuring the incorporation of a fluorescent LPS tracer in the unbound wash fractions. LPS core encapsulation was typically >75%, but surface conjugation was very inefficient at <1%. The incorporation of LPS inside or on the surface of GPs was qualitatively assessed by confocal microscopy (Fig 1). The GPs core labelled with both Dylight 633-OVA and DTAF-LPS showed punctate red and green fluorescence inside the GPs indicating efficient core loading of the respective fluorescently labelled antigens. In contrast, surface loading of DTAF-LPS resulted in broad green fluorescence around the GP particles with only the core loaded Dylight 633-OVA showing punctate fluorescence.

**Fig 2:**
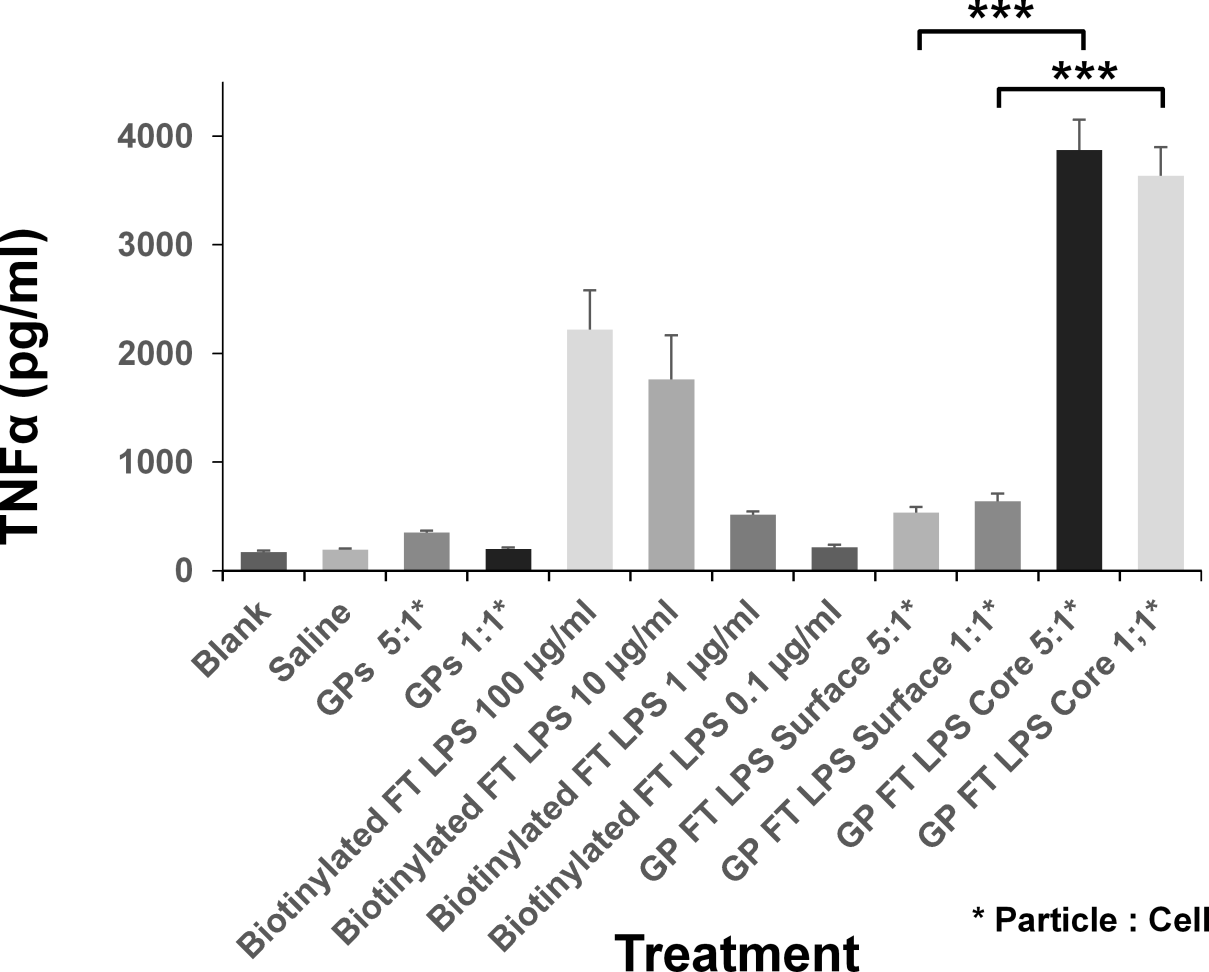
GPs containing core-loaded *F*. *tularensis* LPS are much more efficient at stimulating TNFα secretion as compared to GPs containing surface linked *F*. *tularensis* LPS. Murine BMDCs were stimulated with titrated amounts of biotinylated *F*. *tularensis* LPS (100–0.1 μg/ml), 1 and 5 surface-linked *F*. *tularensis* LPS (GP-Ft LPS Surface) particles/cell, or 1 and 5 GPs containing core-loaded *F*. *tularensis* LPS (GP-Ft LPS Core) particles/cell to produce TNFα. TNFα levels were measured by ELISA. The GP-Ft LPS-core and GP-Ft LPS-surface formulations were tested in duplicate. Error bars show standard deviation. Representative results from 3 independent experiments. ***P<0.001, paired two-tail t-test.

The immunomodulatory potential of GP core loaded and surface linked- LPS formulations was then evaluated by testing their ability to stimulate bone marrow derived dendritic cells (BMDCs) to secrete Tumor Necrosis Factor α (TNFα). The GP core-loaded *F*. *tularensis* LPS formulations were more immunostimulatory in this assay than surface-linked *F*. *tularensis* LPS GP formulations (p>0.001, paired two-tail t-test), or free biotinylated *F*. *tularensis* LPS (Fig 2). Therefore, core-loaded *Francisella* LPS was selected for further evaluation as a component of the GP delivered vaccine.

### *In vivo* dispersal of GPs in mice

**Fig 3:**
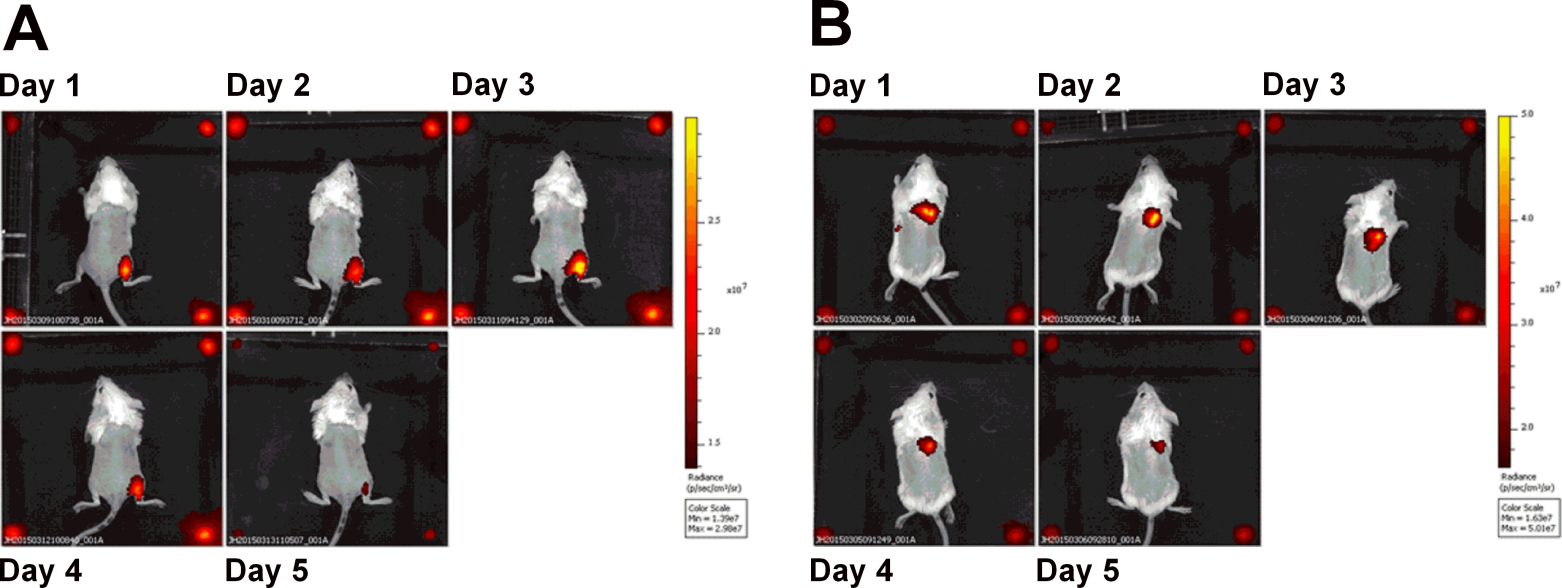
*In vivo* dispersal of GPs in mice. GPs were labelled with Cy7 and used to immunise mice by either intramuscular (Panel A) or subcutaneous (Panel B) routes. After immunisation, mice (n = 3) were imaged daily for 5 days as shown in representative images of same mouse from each administration route (excitation 710 nm-emission 760 nm).

To evaluate the effect of administration route on subsequent dispersal of the GP vaccines, Balb/c mice in groups of 3 were immunized by either the subcutaneous (sc) or intramuscular (im) route with Cy7 labelled GPs. Whole body fluorescence imaging was performed over a period of 5 days post immunisation using an *In Vivo* Imaging System (IVIS). After im administration signal intensity appeared to increase from day 1 to day 3 before declining (Fig 3A), which may reflect an initial quenching effect by the GPs concentrated in the depot at the site of injection. After sc administration a gradual decline in signal intensity was observed, probably as the particles dissipated from the injection site (Fig 3B). There was no signal detected at other bodily locations by IVIS during the study or in individual organs or lymph nodes removed post-mortem on day 5. Signal intensity at the immunisation site after day 5 was comparable for both routes of administration.

### Immunogenicity screening of GP formulated antigens in mice

**Fig 4:**
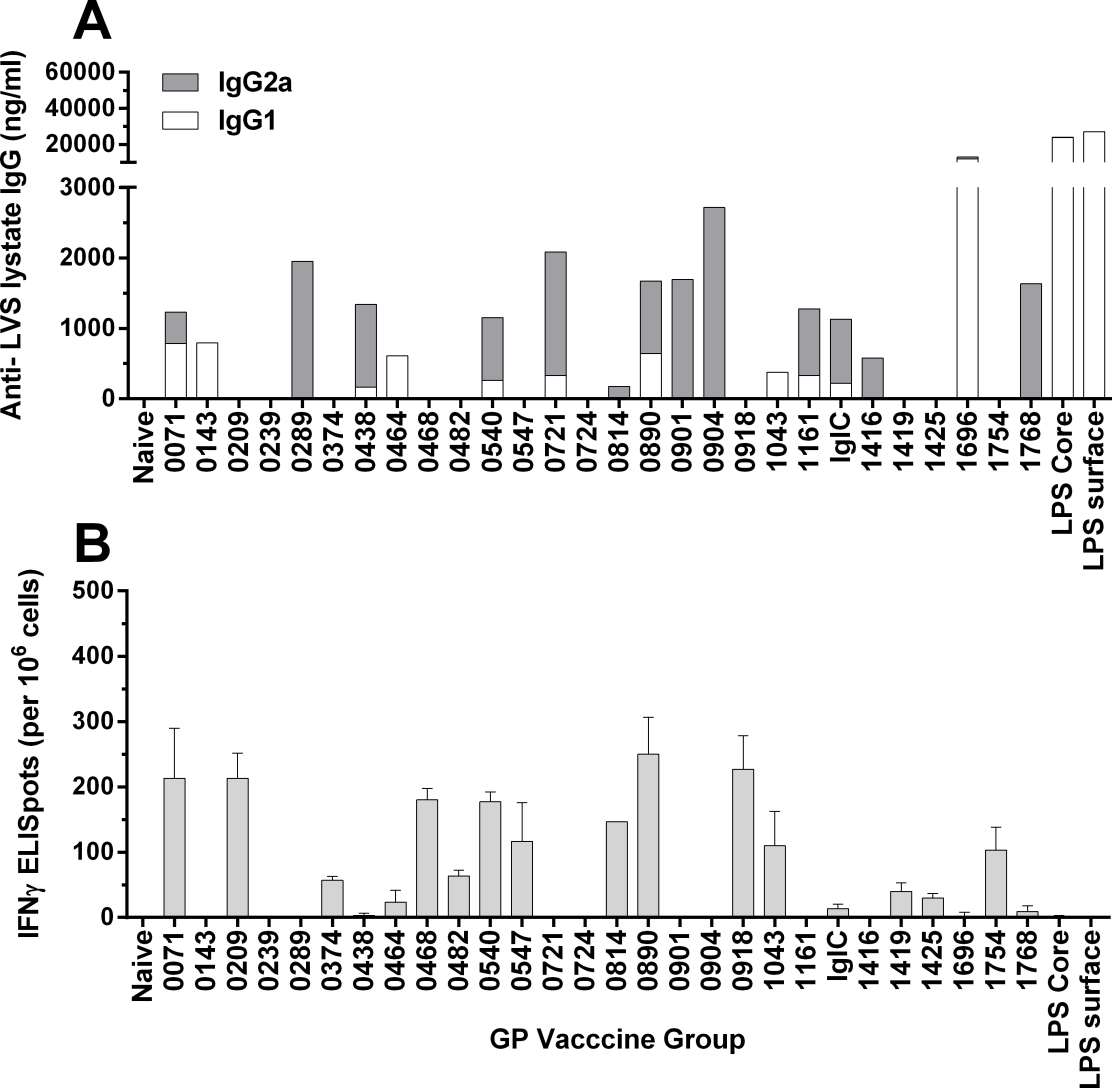
Immunogenicity screen of GP subunit vaccine candidates in mice. Mice (n = 5) were immunised with a panel of GP encapsulated antigens and the development humoral and cellular memory measured. Panel A: IgG1 and IgG2a antibody isotypes recognising antigens in a LVS lysate were measured by ELISA in pooled serum collected 2 weeks after the second vaccination. The mean IgG1 and IgG2a responses (ng/ml) are presented as a stacked bar graph showing the mean response for the pooled serum from 5 mice tested in duplicate for each vaccine group. Panel B: Antigen stimulated expression of IFNγ was measured by ELISPOT. Spleens were harvested from mice 6 weeks after the final vaccination and pooled splenocytes were cultured in triplicate with the antigen corresponding to the immunising GP candidate. Expression of IFNγ was measured by ELISPOT and presented as the mean response (+SEM) per 1x10^6^ splenocytes with the medium-alone response subtracted.

To assist the down-selection of antigen candidates for efficacy evaluation, groups of Balb/c mice (n = 5) were vaccinated on 3 occasions, 2 weeks apart, with the panel of GP packaged antigens shown in Table 1. The ability of the protein and the LPS-core GP vaccines to induce humoral and cellular memory immunity was then assessed. Many of the GP candidates induced a strong antibody response to a crude antigen preparation derived from an LVS-lysate (Fig 4, panel A) demonstrating that the GP vaccines were immunogenic and that humoral immune responses could be induced against the payload antigen. We measured the magnitude of IgG1 and IgG2a isotypes as a high ratio of IgG2a antibody to IgG1 is indicative of Th1-biased immune responses [46], an immune profile which is considered beneficial in controlling *Francisella* infections [47]. The antigens varied in their ability to induce the two antibody isotypes (Fig 4). The LPS packaged GPs induced the strongest antibody response which was exclusively IgG1 biased. To provide a direct measure of antigen-specific T-cell mediated immunity, splenocytes from immunized mice were harvested 6 weeks after the third immunisation and stimulated with the corresponding endotoxin-depleted antigen. The production of Interferon-γ (IFNγ) was measured by Enzyme Linked Immunospot Assay (ELISPOT; Fig 4). Approximately half of the candidates demonstrated an ability to induce a cellular immune response, as evidenced by antigen-specific induction of IFNγ. Since LPS is a T-cell independent antigen, it was not surprising to note that immunisation with GP-LPS particles did not induce an IFNγ response.

To inform our antigen down selection process, we attempted to assess the *in vitro* antimicrobial potential of vaccine induced immunity using a functional killing assay based on previously reported methodology [35]. Splenocytes from immunized mice were stimulated overnight with heat-killed *F*. *tularensis* SchuS4 cells and then co-cultured with *F*. *tularensis* Schu S4-infected J774A.1 macrophage cells. Bacterial growth in macrophages exposed to stimulated splenocytes was compared to growth in macrophages exposed to medium-treated splenocytes and with splenocytes from naïve control mice (S1 Fig). In our hands, this functional killing assay demonstrated a high degree of response variability for immunized animals across all groups including the controls (S2 Fig). Whilst splenocytes from mice vaccinated with FTT0814 induced the strongest magnitude of bacterial control with up to a 22 fold reduction in SchuS4 cultured from infected macrophages, due to assay variability this did not reach significance when compared with splenocytes from naïve control mice (non-parametric ANOVA). Consequently, qualitative assessment of mouse immunogenicity data was used as our primary tool to down-select to seven protein antigen candidates: FTT0071, FTT0289, FTT0438, FTT0814, FTT0890, FTT1043 and IglC. The selection of these candidate antigens was primarily influenced by a combination of the development of antigen specific IFNγ ELISPOT responses and/or the detection of an antibody response, particularly where an IgG2a bias was observed. Since there are currently no robust correlates of protection known for tularemia vaccines, we were interested in selecting candidates that represented a variety of immune response profiles, albeit with a bias toward cell mediated immunity. Although the IglC GP vaccine induced poor immune responses, it was included in our down-selected panel for further evaluation on the basis that when used as a subunit vaccine candidate, IglC has been previously reported to induce partial protection in animals[45].

### Evaluation of down-selected subunit candidates in mice

**Fig 5:**
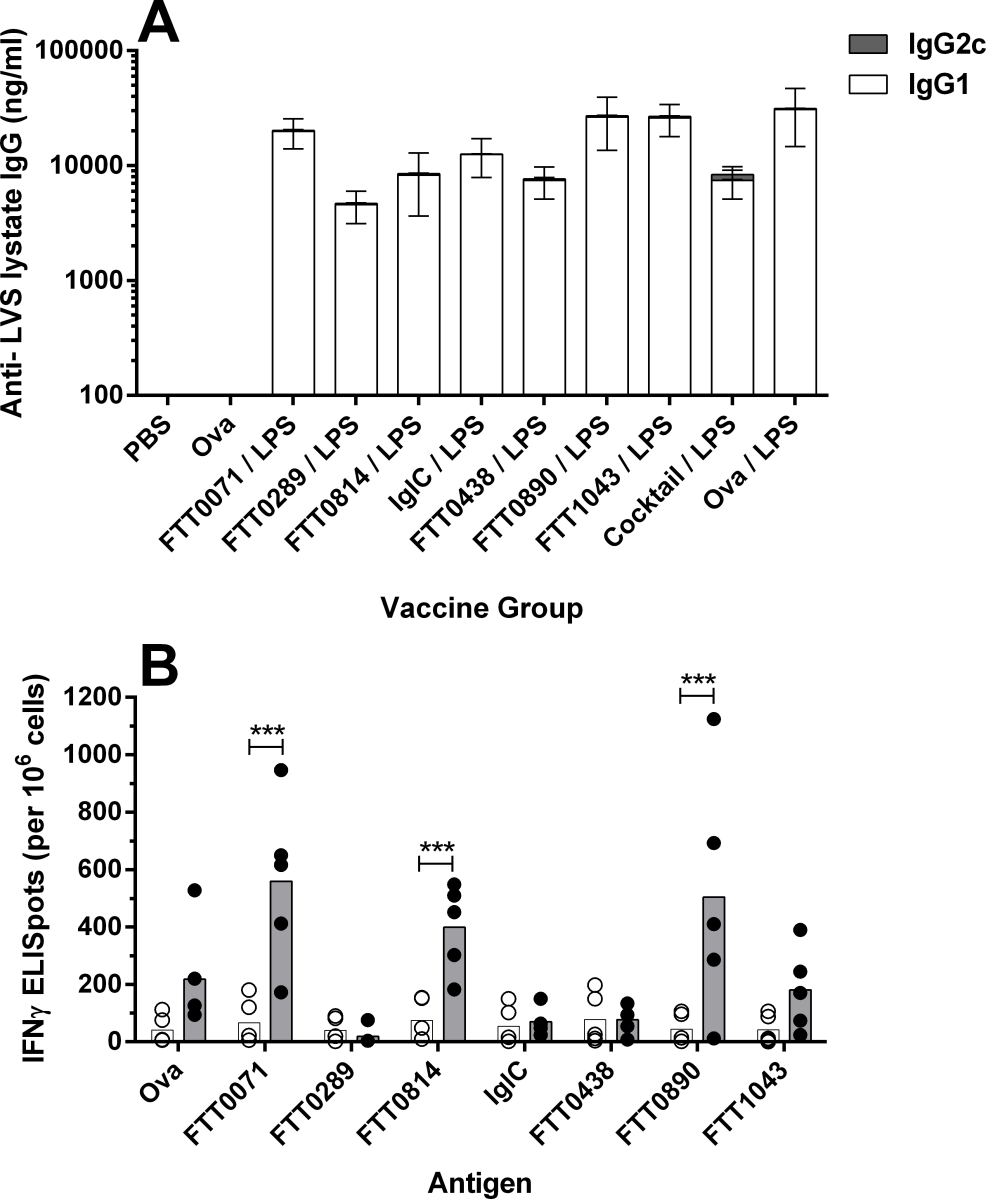
Immunogenicity screen of GP vaccines in mice. C57BL/6 mice (n = 5) were immunised with each of the GP vaccine combinations shown on the x-axis of panel A. The GP “cocktail” vaccine was comprised of a combination of FTT0071, FTT0438, FTT0814 and IglC. Panel A: IgG1 and IgG2c antibody isotypes recognising antigens in a LVS lysate were measured by ELISA in individual serum samples collected 2 weeks after the second vaccination. The mean IgG1 and IgG2c responses (ng/ml) are presented as a stacked bar graph showing the mean response for each vaccine group (+ SEM). Panel B: Antigen stimulated expression of IFNγ was measured by ELISPOT. Spleens were harvested from mice 6 weeks after the final vaccination and splenocytes from individual mice cultured with the antigen corresponding to the immunising GP candidate. Expression of IFNγ was measured by ELISPOT and presented for both medium (white bars / circles) and antigen (black bars/circles) stimulated splenocytes. The circles are the responses for individual mice and the bars are the mean response for the group. Where the antigen-specific response is significantly elevated relative to the medium control, this is indicated (*** p<0.001, t-test with Holm-Sidak multiple comparison correction).

The seven down-selected protein candidates were next evaluated for their ability to induce protective immunity in mice. A C57BL/6 murine model was chosen for further immunological and efficacy evaluation of GP candidates to align approaches used previously to evaluate cryptococcal GP vaccines [48]. Mice were vaccinated with a combination of individual GP-encapsulated protein antigen together with GP-LPS. Co-vaccination with GP-LPS was included with all protein subunit GP candidates to maximise the likelihood of inducing protective immunity. This was on the basis that LPS is the only subunit candidate that has consistently demonstrated protective potential against low virulence strains of *F*. *tularensis* [9–11]. Control groups included a non-vaccinated group, a GP-OVA vector control group and a group vaccinated with GP-LPS. In addition, to assess the potential benefits of an antigen combination vaccine, a “cocktail” comprising GPs individually formulated with FTT0071, FTT0438, FTT0814, IglC and LPS was included in the study. Measurement of IgG1 and IgG2c serum antibodies demonstrated that, with the exception of PBS and GP-OVA controls, all vaccines induced potent antibody responses that recognized antigens in an LVS-lysate (Fig 5, panel A). The IgG2c subclass is associated with Th1 responses in the C57BL/6J mouse [49]. A predominantly IgG1 isotype was observed which persisted for at least 70 days (S3 Fig). However, this antibody response most likely reflects the inclusion of LPS in the vaccine formulation, LPS being a sero-dominant IgG1 inducing antigen, as demonstrated in the previous mouse immunogenicity screening experiments.

A cohort of vaccinated mice (n = 5) were culled 6 weeks after the third vaccination to assess T-cell immunity. FTT0071, FTT0814 and FTT0890 GP-vaccines induced the strongest antigen-recall IFNγ ELISPOT responses in splenocytes cultures (Fig 5). The dominance of these candidates as T-cell antigens was consistent with responses observed previously in Balb/c mice (Fig 4). Recall responses to all candidate antigens were also assessed using splenocytes isolated from mice that had been vaccinated with the GP cocktail of FTT0071, FTT0438, FTT0814, IglC and LPS. In this GP-Cocktail group, only FTT0071 and FTT0814 primed for strong IFNγ memory responses (S4 Fig).

A broad range of cytokine responses was measured in 72-hour antigen stimulated splenocytes cultures using a cytokine multiplex bead assay (CBA) (S5 Fig). This assay measured Interleukin-6 (IL-6), IL-10, Monocyte Chemotactic Protein-1 (MCP-1), IFNγ, TNFα and IL-12. The CBA data confirmed FTT0071, FTT0814 and FTT0890 as the most potent inducers of IFNγ responses. These antigens also induced strong IL-6 and TNFα responses, both of these being potent proinflammatory cytokines. The pattern of responsiveness observed for MCP-1 was less consistent compared with the other pro-inflammatory cytokines. However, given that elevated MCP-1 responses were observed even in the antigen-unstimulated cultures, the antigen specificity of MCP-1 responses is uncertain. IL-12 responses were also measured but responses were below the assay detection sensitivity for all vaccine/antigen combinations (data not shown). FTT0814 stimulated the strongest and most consistent IL-10 response.

To assess the efficacy of these GP vaccine combinations, 6 weeks after the final vaccination, groups of 5 mice were challenged with a low dose of *F*. *tularensis* Schu S4 via the ip route. The challenge dose was confirmed to be 7 CFU. However, we failed to observe any protective efficacy in this study for any of the GP vaccine candidates (S6 Fig). Our ability to observe a protective effect was limited by the observation that 2/5 of the non-vaccinated control mice did not succumb to a lethal infection as we would have expected. This was presumed to be a consequence of aiming to deliver such a low challenge dose, a dose chosen due to the extremely high susceptibility of mice *to F*. *tularensis* infections. As an alternative to attempting to refine the challenge model in mice, we chose to transition efficacy evaluation to the alternative inbred Fischer 344 (F344) rat model. Due to the lower susceptibly of F344 rats to *F*. *tularensis* infections, the model provides the potential to increases the discriminatory power to measure protective responses.

### Computational analysis

Detailed analysis of the 7 lead antigens, and comparison to the larger panel of proteins in Table 1, indicated that down-selection based on immune responses in mice had indeed resulted in selection of antigens predicted to be immunogenic, and also in eliminating several of the original 30 which have a high content of self-like T cell exposed motifs predicted as likely to elicit an immunosuppressive response [36,38]. These proteins were 1740 (FTT_0918), Tep3 (FTT_1754), KatG (FTT_0721) for MHC I, and 1740 (FTT_0918), Tep3 (FTT_1754), 2227 (FTT_1768) and Tep15 (FTT_0540) for MHC II. These proteins are each in the top 10% within the *F*. *tularensis* proteome in their content of common T cell exposed motifs (TCEM) per 100 amino acids, where common is defined as a motif occurring at greater than 1 in 1024 immunoglobulin variable regions.

Relative to these proteins, the content of self-like TCEM in the lead 7 proteins was much less. IglC carries within peptide QGSLPVCCAASTDKG (amino acid index position 187) an amino acid motif S~~V~C~AS which is a T cell exposed motif found in 1 in 16 immunoglobulin variable regions and thus a very common occurrence which would encounter a large cognate T cell population. This peptide is predicted to be bound at high affinity by most DQA and DQB alleles, and less strongly by murine allele H2-IAd. FTT0071 has high frequency TCEM at two positions (amino acid index position 23 LPVYSPSLG and 233 QNASTSTVR). These peptides comprise motifs found in at least 1 in 32 immunoglobulin variable regions. However, their impact would be limited to the MHC alleles to which they bind at high affinity. This is predicted, in both cases, to include several human HLA but not murine alleles H2Kb or H2Kd.

With the exception of FTT0890, the selected proteins each have strong predicted B and T cell epitopes. In FTT0890, predicted MHC binding is confined to the transmembrane domain region of the protein.

**Fig 6:**
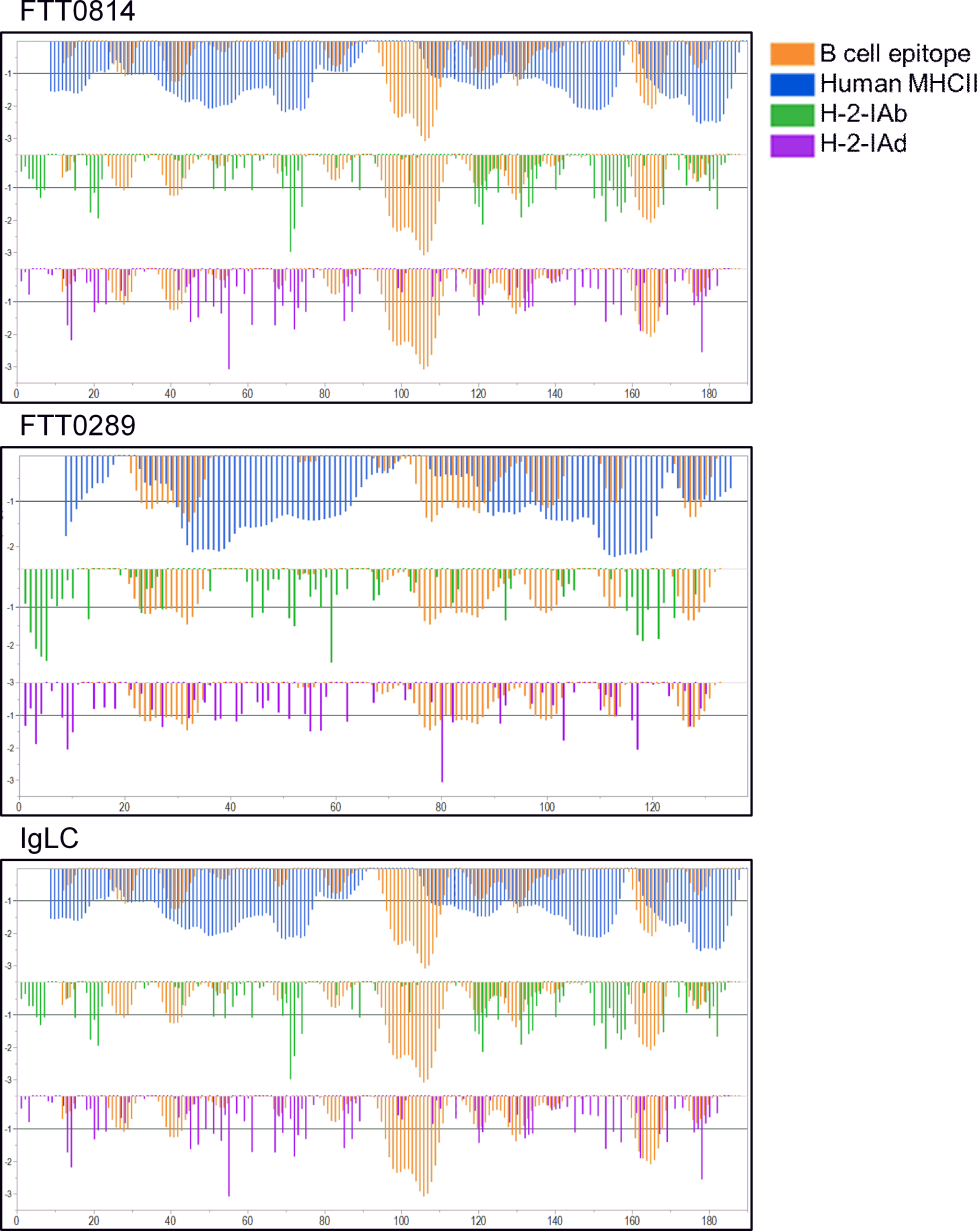
Comparison of human and murine MHC II binding patterns and B cell linear epitopes. For each of the three proteins FTT0814, FTT0289 and IglC, the predicted MHCII binding affinity of sequential 15-mer peptides is shown on the X axis, positioned by index amino acid position of each 15-mer. Within each panel, the top tier shows the average predicted binding to human MHC II, averaged for DRB alleles (blue). The middle tier shows the predicted binding to H-2-IAb for C57Bl/6 mice (green). The bottom tier shows predicted binding to H-2-IAd for Balb/C mice (purple). Overlayed onto each of the human MHCII, H-2-IAb and H-2-IAd binding predictions are the probable B cell linear epitopes (orange). Y axis units for MHC binding are standard deviation units below the mean of the natural log binding affinity (lnIC_50_) for that protein; and inverted standard deviation of linear B cell epitope probability (inverted).

The 7 proteins differed in the degree to which predicted T cell epitopes for the two mouse strains examined would predict a broad human response. However, three of the antigens, FTT0289, FTT0814, and IglC do have predicted murine responses that closely resemble the overall human pattern as shown for MHC II (Fig 6). The 7 lead proteins are members of FIGfams [39] which are highly conserved across 100 isolates of *F*.*tularensis* examined.

### Immunological evaluation of GP vaccine candidates in F344 rats

**Fig 7:**
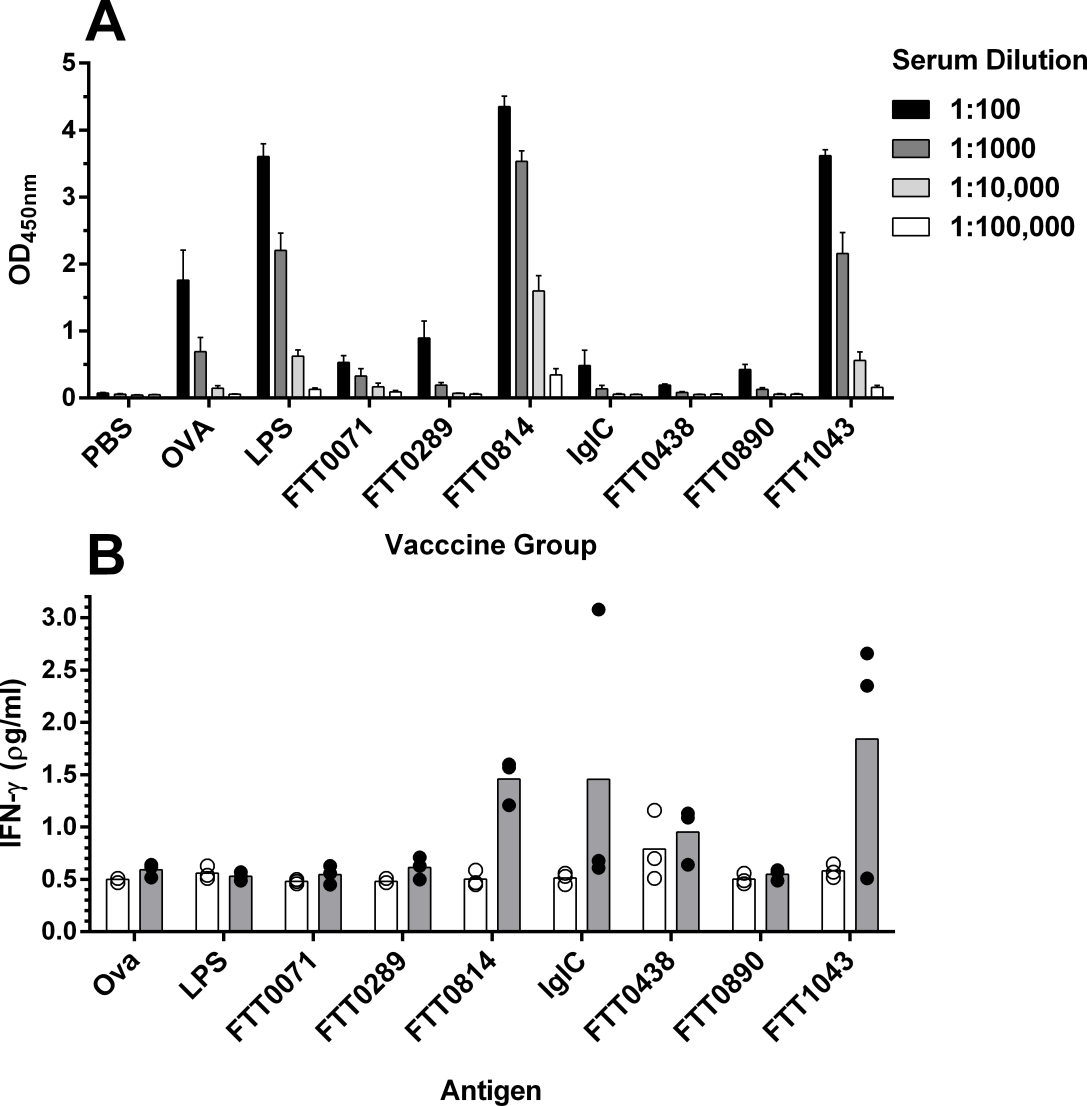
Immunogenicity evaluation of GP vaccine candidates in F344 rats. F344 rats (n = 3) were immunised with each of the GP vaccine combinations shown on the x-axis of panel A and immune responses measured 2 weeks after the third vaccination. Panel A: Total IgG antibody recognizing the antigen corresponding to that in the immunizing GP vaccine was measured in serum by ELISA. The response (OD450nm) for the individual rats in each group is shown for the range of indicated serum dilutions. Each bar represents the mean response (+SEM). Panel B: Antigen stimulated expression of IFNγ was measured in PBMCs. PBMCs from individual rats were cultured with the antigen corresponding to the immunizing GP candidate and expression of IFNγ measured by ELISA. PBMC antigen recall responses are reported in the non-vaccinated PBS immunised group (white bars / circles) and in the corresponding GP-antigen immunised groups (black bars/circles). The circles are the responses for individual rats and the bars are the mean response for the group.

F344 rats are susceptible to *F*. *tularensis* infection but unlike in mice, LVS can induce protective immunity even against high virulence strains [16,18] and could thus be considered a more appropriate rodent model for efficacy evaluation of *F*. *tularensis* vaccine candidates. Therefore, we transitioned the evaluation of our GP candidates into a F344 rat model. Initially, we performed a pilot immunogenicity study to determine the hierarchy of immunological responsiveness of the seven down-selected *F*. *tularensis* antigens in rats. Groups of rats (n = 3) were immunized on 3 occasions, 2 weeks apart, with each of the GP packaged antigens FTT0071, FTT0289, FTT0438, FTT0814, FTT0890, FTT1043, IglC and LPS. Unvaccinated and GP-OVA groups were included as controls. Immune responses were measured in blood taken 2 weeks after the third vaccination (Fig 7). The only *Francisella* antigens that induced strong serum IgG responses were LPS, FTT0814 and FTT1043. All GP vaccinated rats developed an IgG response to the OVA marker protein indicating that where no response to GP immunized *Francisella* antigens was observed, this was not as a consequence of deficiencies with the immunisation protocol (S7 Fig). Induction of cell mediated immunity was assessed by measurement of IFNγ from antigen stimulated PBMCs. IFNγ responses were generally low. IFNγ antigen recall responses were most consistently elevated for the GP candidates FTT0814 and FTT1043. Interestingly, FTT0071 failed to induce detectable IFNγ or IgG responses in GP-FTT0071 vaccinated rats despite this antigen being strongly immunogenic in mice. Whilst immunisation with FTT0438 did not result in a detectable IFNγ response when splenocytes were re-stimulated with FTT0438 protein, a response was detected in all 3 rats when re-stimulated with LVS lysate antigen preparation (S8 Fig). Therefore, taken together, FTT0814, FTT1043 and FTT0438 were selected for further efficacy evaluation in the F344 rat model.

### Efficacy evaluation of GP vaccines in F344 rats

Based on the immunogenicity data, FTT0814, FTT0438 and FTT1043 were taken forward for evaluation in a F344 rat aerosol challenge model. We adopted a strategy of immunising rats with the individual GP-encapsulated protein antigen together with GP-LPS. As previously, this strategy was adopted to maximise the likelihood of inducing protective immunity through the inclusion of a known partially protective antigen. One group of rats was vaccinated with a cocktail of the individually formulated GP encapsulated antigens FTT0814, FTT0438 and FTT1043 and LPS. Controls groups included a sham-vaccinated group (PBS), a GP-OVA vector control group and a group vaccinated with GP-LPS alone. In addition, one group of rats was vaccinated with 1x10^7^ CFU of LVS. Vaccine induced immunity was measured in tail bleed samples collected 2 weeks after the third GP vaccination and prior to challenge (S9 Fig). Consistent with the previous immunology study, of the 3 protein antigens, only FTT0814 and FTT1043 induced detectable IgG responses. In the rats immunized with the GP cocktail of antigens, FTT0814 elicited the strongest and most consistent IgG response. Little or no antigen stimulated IFNγ responses were detected in PBMCs isolated from rats immunized with the individual protein / LPS vaccine combinations. However, in the GP cocktail immunized mice, elevated IFN responses to FTT0814 were detected (S9 Fig).

**Fig 8:**
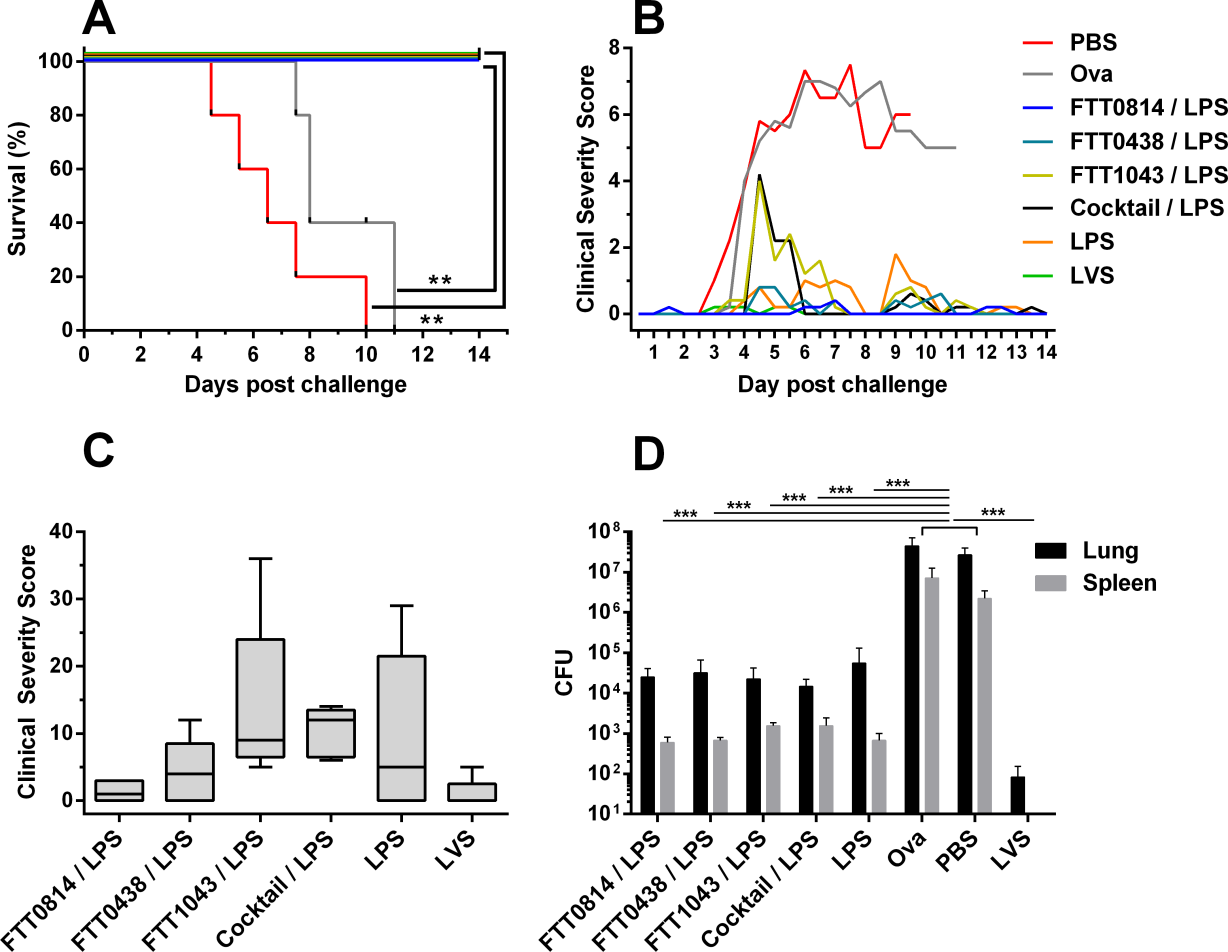
GP-based vaccines protect F344 rats from a lethal aerosol challenge of *F*. *tularensis*. F344 rats were vaccinated with GP vaccines combinations, LVS or PBS and then challenged with an aerosol of *F*. *tularensis*. Panel A: Rats were monitored for 14 days and culled if they reach predefined humane endpoint criteria as presented on the Kaplan-Meier survival curve (p<0.01 Log rank test). Panel B: Clinical signs were assessed twice daily and an accumulative clinical score was calculated, presented as the mean score for each group during the 14 day post infection period. Panel C: Accumulative total severity score for the entire 14-day post infection period for rats in vaccine groups that survived the lethal challenge (the box represents the interquartile range, the whiskers the range and the horizontal line the median. Panel D: Bacterial enumeration of *F*. *tularensis* in lungs and spleen of rats (mean CFU/tissue with SEM). For PBS and Ova groups, tissues were processed on the day that animals met their humane endpoint, for all other groups tissues were processed at day 14 post infection (p<0.001, 2 way-ANOVA with Sidak’s post analysis multiple comparison test).

All rats were challenged with a calculated retained dose of 1.6 x 10^3^ CFU *F*. *tularensis* SchuS4 delivered by the aerosol route. All PBS sham-immunized and GP-OVA vector control rats succumbed to a lethal infection between days 4–11 post infection (Fig 8A). An apparent delay in the time to death between the PBS and GP-OVA groups did not quite reach significance (p = 0.0551, Log rank test). All GP vaccine groups that received GP-antigen and/or GP-LPS survived the aerosol challenge of *F*. *tularensis* and this effect was significant when compared to the survival of the PBS and GP-OVA control groups (p<0.01, Log rank test). The scoring of clinical signs in the rats identified that transient clinical indications of infection were observed in many of the rats that survived the challenge. These clinical signs were particularly apparent between days 4–6 post-infection (Fig 8B). To explore this further, a comparative analysis of the clinical scores over the 14 day period was performed. However, whilst transient clinical signs were observed less frequently in the rats in groups vaccinated with either LVS or the GP FTT0814 / LPS combination, the differences between groups were not significant (p>0.05, Kruskal-Wallis nonparametric test with Dunn’s post analysis multiple comparisons). All surviving rats were culled at day 14 post-challenge and total bacterial loads enumerated in lungs and spleens. *F*. *tularensis* was cultured from the lung and spleen from all GP-vaccinated rats, although at significantly reduced levels compared with either PBS or GP-OVA groups (p<0.001, 2 way-ANOVA with Sidak’s post analysis multiple comparison test). The LVS vaccinated group demonstrated enhanced bacterial clearance with an absence of detectable *F*. *tularensis* in the spleens, and significantly reduced bacterial burden in the lung compared with all of the GP-vaccine groups (p<0.001, 2 way-ANOVA with Sidak’s post analysis multiple comparison test). The rats vaccinated with LVS were also the only group where individual rats continued to steadily gain weight after aerosol challenge (S10 Fig).

## Discussion

The majority of tularemia vaccine evaluation studies have been performed in the mouse. The mouse model has been used to demonstrate efficacy of live attenuated *F*. *tularensis* strains (reviewed in 8) and has provided a valuable tool to assess tularemia vaccines. It is also a good model for antigenicity screening as the responses are reproducible, reagents are available and the responses to LVS immunisation have been well-characterised (reviewed by 36). However, this is a highly susceptible model, especially to respiratory challenge. This may limits its discriminatory power as model to assess vaccine mediated protection, particularly with respects to identifying promising sub-optimal vaccines (18). In view of the strengths and limitations of the mouse model, we undertook a preliminary immunogenicity screen of our antigens delivered by GPs in the mouse model, but transitioned to the F344 rat model for evaluation of protection.

The only antigen reported to induce even partial protection as a subunit in the mouse model is LPS [9–11]. *F*. *tularensis* LPS is unusual in that unlike LPS from most other Gram negative bacteria, it is a poor TLR4 ligand [50], but as an antigen, it primes for strong antibody responses [51]. In order to compare these differing roles of LPS in the immune response to *F*. *tularensis*, we reasoned that LPS might need to be present on the surface of GPs to facilitate TLR signalling, as opposed to being delivered in GP cores as an immunising antigen. To this end, a strategy was developed which allowed for surface linking of *F*. *tularensis* LPS onto the surface of GP particles instead of the standard core loading of the antigen. GPs containing core-loaded *F*. *tularensis* LPS were more efficient at stimulating TNFα secretion by murine BMDCs as compared to GPs containing surface-linked LPS. Previously, synergies had been reported between TLRs and the β1,3 glucan in the GPs [52], but it appears that the delivery of *Francisella* LPS intracellularly dominates the cellular response. *Francisella* species have tetra-acylated lipid A unlike the hexa-acylated species of enteric bacteria [53] allowing evasion of caspase-11 recognition [54]. It is generally accepted that neither lipid A nor LPS from *F*. *tularensis* interacts with TLR4, or other TLRs [50,55,56] with the exception of heterodimers including TRL2 [57]. Cytosolic, intracellular *Francisella* can be recognized by intracellular receptors including AIM2, pyrin and NLRP3, resulting in inflammasome activation [58–63]. Therefore the observed enhanced LPS bioactivity by core versus surface loading of GPs may be due to intracellular delivery and subsequent interaction with Nod-like receptors. This in turn may affect the immune response to concomitantly delivered protein antigens [64].

The proteins delivered in GP vaccines were immunogenic: antibody could be detected against the loaded antigens and some candidates also induced cell mediated immune responses. The injection of the GP vaccines resulted in a depot effect at the site of immunisation lasting at least 5 days, which may also explain the adjuvant effect of GP delivery, in addition to the interaction of β1,3-D-glucans with the Dectin-1 receptor to trigger proinflammatory cytokine secretion observed previously [29,65].

The immunological analysis of protein candidates in mice allowed an initial down-selection from 30 proteins to 7 proteins, a more practical number of candidates for more detailed immunological and efficacy evaluation. These lead antigens were analysed using a more comprehensive computational approach (S1 Table). This approach identified 6 of the 7 antigens as likely good immunogens with strong B cell and T cell epitopes, although two (IglC and FTT0071) may elicit a more evasive or suppressive response for some human alleles due to the presence of very common T cell exposed motifs. It further indicated that the 7 proteins are highly conserved across many isolates of *F*. *tularensis*. When murine MHC allele binding was compared to human alleles, the predictive value of the murine response was better for FTT0289, FTT0814, and IglC than for the other proteins. In the absence of training sets to establish predictive algorithms for Fischer rats, projections cannot be made as to how murine and human predicted responses compare to this species.

We attempted to assess protective responses to these seven GP encapsulated proteins, delivered together with LPS, in mice challenged with a low ip dose of *F*. *tularensis* SchuS4. We were unable to demonstrate protection for any of the candidates, but this was largely a consequence of the difficulty of delivering a very low challenge dose. This served to highlight the challenges of the mouse model for efficacy evaluation of *F*. *tularensis* vaccine candidates.

The F344 rat has been proposed as a more appropriate model for *F*. *tularensis* vaccine efficacy testing as it is more resistant to tularemia than the highly susceptible mouse model and overall the pathogenesis of respiratory tularemia in the rat model appears to replicate tularemia in humans [66]. Killed whole cell formulations were able to induce protective immune responses in rats against systemic challenge [67] and LVS immunisation was protective against aerosol challenge [68]. The efficacy of the GP vaccines in inducing protective immune responses was therefore evaluated in the Fischer 344 rat. An immunological bridging study was first undertaken to determine the hierarchy of immunological responsiveness of the seven down-selected *F*. *tularensis* antigens in rats. Responses to the carrier protein, OVA, included in each vaccine were lower than seen in the mouse, but it has previously been reported that Fischer rats are ‘low immunological responders’ to OVA even when compared with other rat strains such as Wistar and Sprague-Dawley rats [69]. Whilst the hierarchy of immune responsiveness in mouse and rat models was largely overlapping, FTT0071 was a notable exception. Whilst this antigen was immunodominant with regards to IgG and IFNγ responses in mice, it induced poor responses in rats. The two lead vaccines containing FTT0814 or FTT1043 and LPS, and a less immunogenic vaccine containing FTT0438 protein and LPS, were taken into an in-depth immunology study and an evaluation of protection. Consistent with the previous immunology study, evaluation of pre-challenge responses in rats vaccinated with the FTT0814-based GP vaccine demonstrated the strongest and most consistent antigen specific IgG response and the strongest T-cell mediated IFNγ responses. We subsequently demonstrated that these GP encapsulated *F*. *tularensis* antigen combinations protected against an otherwise lethal aerosol challenge of *F*. *tularensis* SchuS4. The ability of a subunit vaccine to protect against the aerosol delivery of a fully virulent strain of *F*. *tularensis* has not previously been reported. In the challenge experiment, it was not possible to determine from survival results whether there was a contribution to protection from the protein antigens, as vaccination with GP-LPS alone was sufficient to provide protection at the challenge dose used. In many animals, including humans, the primary response is directed against LPS, and thus the rat seems to model the human infection and protection [17,70,71]. Furthermore, given that LPS is a T-cell independent antigen, which is predominantly associated with the generation of strong antibody responses, this highlights the importance of antibody in protective immunity to *F*. *tularensis* infection. However, it was noted that the rats immunized with GP-LPS vaccine or vaccines containing GP-FTT0438-LPS developed stronger and more persistent transient clinical signs of disease compared with the animals receiving the GP-FTT0814-LPS vaccine or the LVS vaccinated rats. Although not conclusive, this is suggestive that FTT0814 may supplement the protection induced by LPS and warrants further evaluation. This is also consistent with the computational analysis which showed FTT0814 to be a promising immunogen for both rodents and humans. The LVS vaccine was more protective than the GP-based vaccines with respects to enhanced bacterial clearance and improved post-infection weight gain in vaccinated rats. This observation indicates that the model is sufficiently sensitive to determine differences in levels of protection between candidates, an important consideration for assessing further optimisation of GP-based vaccination strategies.

Therefore, in summary, we report the first subunit vaccine which protects against a robust lethal aerosol challenge of *F*. *tularensis* in an inbred small animal model. Protection was largely mediated by the delivery of *Francisella* LPS by GPs, but we have also identified a promising complementary protein antigen. The computational analysis reported here can be applied to the entire *F*. *tularensis* genome to identify other promising candidates which may further supplement protection. This opens further the possibility of developing a subunit vaccine to prevent tularemia, and demonstrates the broad immunological responses that can be induced by GP technology. It also highlights the importance of using an appropriate animal model for efficacy studies versus immunogenicity screens.

## Supporting information

S1 Fig GP antigen loading efficiency. A. Loading efficiency for GPs was calculated by including rhodamine-labelled OVA in each formulation. The unbound (UB) fluorescence in relative fluorescence units (RFU) was subtracted from the RFU of the fluorescent load to calculate % Loading = (FL-UB/FL) x 100. B. SDS-PAGE analysis of vaccine. * assumes all unbound material is loaded in the GPs. M: Protein ladder; 1: OVA (5 μg); 2: FTT0814 (5 μg); 3: unbound vaccine supernatant fraction (25 μl); 4: protein from GP vaccine pellet fraction (25 μl) were analysed by 10% SDS-PAGE to demonstrate antigen identity (green boxes) and loading as demonstrated by the absence of loaded proteins in the unbound supernatant fraction. Extracting antigens that were loaded in urea by boiling in sample buffer is not efficient as evidenced by the light antigen band in lane 4. The gel image provided is a composite image of the samples separated on SDS-PAGE gels under identical run conditions.
(TIF)

S2 Fig Macrophage/splenocyte killing assay. Mice (n = 5) were immunised with a panel of GP encapsulated antigens. Splenocytes were harvested, pooled by vaccine group and stimulated in triplicate with either medium, PMA/ionomycin or heat killed *F*.*tularensis* SCHU S4 (ND = not done). Stimulated splenocytes were co-cultured with J774.2 murine macrophages which had been infected with SCHU S4 at a multiplicity of infection of 10. The splenocytes and infected cells were co-cultured in triplicate for 48 hours and intracellular bacteria enumerated in triplicate. Panel A presents the mean CFU/ml (+ SEM) for the triplicate cultures using splenocytes which had been pre-stimulated with either medium, PMA/ionomycin or heat killed cells (HK). For logistical reasons, it was necessary to process immunised mice in 4 separate studies as indicated on the figure. Panel B presents the data as a ratio of the number of bacteria enumerated in *F*. *tularensis* antigen-stimulated (HK) splenocyte co-cultures compared with the medium-stimulated splenocyte co-culture, termed the bacterial control index (presented as mean of triplicate cultures + SEM).
(TIF)

S3 Fig IgG antibody responses in mice 70 days post immunisation. C57BL/6 mice (n = 5) were immunised with each of the GP vaccine combinations shown on the x-axis. The GP “cocktail” vaccine was comprised of a combination of FTT0071, FTT0438, FTT0814 and IglC. IgG1 and IgG2c antibody isotypes recognising antigens in a LVS lysate were measured by ELISA in serum collected 70 days after the first of 3 vaccinations, Serum samples from individual mice were tested in duplicate and IgG1 and IgG2c responses (ng/ml) presented as a stacked bar graph showing the mean response for each vaccine group (+ SEM).
(TIF)

S4 Fig IFNγ ELISPOT responses in mice vaccinated with the GP cocktail. C57BL/6 mice (n = 5) were vaccinated with the GP cocktail containing FTT0071, FTT0438, FTT0814, IglC and LPS. Mice were culled 6 weeks following the final booster vaccination and IFNγ responses measured in splenocyte cultures stimulated with each of the antigens shown on the x-axis. Responses for individual animals are shown as circles and the mean response for the group is shown as the corresponding grey bars.
(TIF)

S5 Fig CBA cytokine response in mice vaccinated with GP subunit vaccines. C57BL/6 mice (n = 5) were immunised with combinations of GP formulated LPS and each of the following candidate antigens; FTT0071, FTT0289, FTT0438, FTT0814, FTT0890, FTT1043 and IglC. Mice were culled 6 weeks following the final vaccination and cytokine responses measured in splenocyte cultures stimulated with each of the respective protein antigens. Cytokines were detected using a CBA multiplex assay and are shown for IL-6, IL-10, MCP-1, IFNγ and TNFα in each of the indicated panels. Responses for individual animals are shown as circles and the mean response for the group is shown as the corresponding grey bars.
(TIF)

S6 Fig Efficacy evaluation of GP vaccines in mice. C57BL/6 mice (n = 5) were vaccinated with GP vaccines combinations shown in the legend of the figure and then challenged with a low dose (~7CFU) of *F*. *tularensis* SCHU S4 via the ip route. Mice were monitored for 14 days and culled if they reach predefined humane endpoint criteria as presented on the Kaplan-Meier survival curve.
(TIF)

S7 Fig Ova serum IgG responses in GP vaccinated F344 rats. F344 rats (n = 3) were immunised with each of the GP vaccine combinations shown on the x-axis and IgG responses measured 2 weeks after the third vaccination. Total IgG antibody recognizing Ova was measured in serum by ELISA. The response (OD450nm) for the individual rats in each group is shown for the range of indicated serum dilutions. Each bar represents the mean response (+SEM).
(TIF)

S8 Fig LVS-lysate stimulated IFNγ responses in GP vaccinated F344 rats. F344 rats (n = 3) were immunised with each of the GP vaccine combinations shown on the x-axis. PBMCs were isolated 2 weeks after the third vaccination and stimulated with a LVS-lysate crude antigen preparation. IFNγ in 72 hour culture supernatants was measured by ELISA. The open circles are the responses for individual rats and the bars are the mean response for the group. Where a significant difference between responses in the unvaccinated (PBS) and GP vaccinated groups was observed, this is indicated (* p<0.05, non-parametric Kruskal-Wallis analysis with Dunn’s multiple comparison post analysis test).
(TIF)

S9 Fig Pre-challenge immune responses in vaccinated F344 rats. Immune responses were measured in blood samples collected 2 weeks after the third vaccination with the following GP vaccines; LPS, FTT0814+LPS, FTT0438+LPS, FTT1043+LPS and a cocktail of FTT0814, FTT0438, FTT1043 + LPS. Panel A: Antigen specific serum IgG responses in each of the groups vaccinated with individual protein or LPS GPs. The triangles are the response for individual rats and the bar is the mean response for the group. Panel B: Serum IgG responses to each of the component GPs antigens in rats immunised with the GP cocktail (data reporting as per panel A). Panel C: Antigen stimulated expression of IFNγ was measured in PBMCs from rats vaccinated with individual protein or LPS GPs. PBMCs from individual rats were cultured with the antigen corresponding to the immunizing protein GP candidate and expression of IFNγ measured by ELISA. Antigen recall responses are reported in the non-vaccinated PBS immunised group (white bars / circles) and in the corresponding GP-antigen immunised groups (black bars/circles). The circles are the responses for individual rats and the bars are the mean response for the group. Panel D: Antigen stimulated PBMC IFNγ responses were measured for each of the component GPs antigens in rats immunised with the GP cocktail (data reporting as per panel D). Where a significant difference between responses in the unvaccinated (PBS) and GP vaccinated groups was observed, this is indicated (*p<0.05, multiple t-test corrected for multiple comparisons using the Holm-Sidak method).
(TIF)

S10 Fig Weight change in rats after aerosol challenged with *F*. *tularensis*. F344 rats were vaccinated with GP vaccines combinations, LVS or PBS and then challenged with an aerosol of *F*. *tularensis*. The percentage weight change for individual rats in each of the 8 treatment groups is presented in each of the 8 panels. In the PBS and GP-Ova groups, rats were culled when they reached pre-defined humane endpoints of clinical severity or when the weight loss reached 15%.
(TIF)

S1 Table Computational *in silico* methods applied to predict immunogenicity of peptide epitopes. (DOCX)
