# Supplementary material for: Protection induced by a Francisella tularensis subunit vaccine delivered by glucan particles
Source: PLoS One. 2018 Oct 8;13(10):e0200213. doi: 10.1371/journal.pone.0200213 (PMC6175290; doi:10.1371/journal.pone.0200213)
Supplement: S1 Table — (DOCX) [file pone.0200213.s011.docx]

**S1 Table**. Computational *in silico* methods applied to predict immunogenicity of peptide epitopes.

| **Immunological Metric** | **Method** | **Prediction** |
| --- | --- | --- |
| MHC I affinity  9-mer | 1. Neural Network Ensembles trained on binding data 2. sliding window of 9-aa indexed by 1 amino acid 3. 20 HLA-A 4. 17 HLA-B 5. 6 murine | 1. LN (IC_50_) 2. std dev LN(IC_50_) (ave = 0.5 LN (IC_50_) ) 3. standardized affinity within protein or proteome (enables using additivity of variance) 4. relative binding probability thresholds for each peptide |
| MHC II affinity  15-mer | 1. Neural Network Ensembles trained on binding data 2. sliding window of 15-amino acids indexed by 1 amino acid 3. 16 DR, 6 DP, 6DQ 4. 3 murine | 1. LN (IC_50_) 2. std dev LN(IC_50_) (ave = 0.5 – 0.7 LN (IC_50_) ) 3. standardized affinity within protein or proteome (enables using additivity of variance) 4. relative binding probability thresholds for each peptide |
| MHC + MHC II cross presentation | MHC I + MHC II simultaneous binding to peptides in a protein | Number of high affinity MHC I 9-mers within each high affinity MHC II 15-mer |
| Linear B-cell epitope  8-mer | 1. Neural network trained on B-cell epitopes 2. Sliding window of 8 amino acids indexed by 1 aa | 1. probability of B cell binding standardized within protein 2. relative probability among peptides |
| Cathepsin cleavage  Human cathepsin B, L and S | Neural Network Ensembles trained on large proteomic cleavage database mass spectrometry of cleaved peptides for the enzymes. | Two independent predictions  probability of cleavage + probability of non-cleavage between aa4 and aa5 (P1P1’) of an octomer |
| Combined cathepsin cleavage + MHC I + MHC II binding affinity | Combination of 3 prediction method outputs | Probability of excision of various length peptides between 15-21 amino acids in length for MHC II and exactly 9 amino acids. |
| T-cell exposed motif (TCEM) relative to normal human repertoires | Frequency comparison to database for:  continuous pentamer within a bound MHC I 9-mer  two discontinuous pentamers (9-mer core of a bound MHC II 15-mer) | 1. Specific motifs exposed to T cell by peptide bound in MHC 2. Frequency relative to IGHV germline and somatic mutation 3. Frequency relative to human proteome 4. Frequency relative to GI microbiome |
| Combination of MHC I and MHC II binding by allele in combination with TCEM T-cell exposure and cathepsin cleavage | Graphical / interactive combination of 5 different prediction outputs | Interactive graphical platform for evaluation Treg potential based on combination of:   1. binding predictions and 2. TCEM frequency data using additivity of variance |
| Protein topology | 1. Neural Network Ensembles 2. Slider, graphical interface 3. compared to Web references | 1. Amino acids of protein comprising extra-cellular domain 2. Amino acids of protein comprising intra-cellular domains 3. Amino acids of protein comprising trans-membrane domains 4. Signal peptides cleavage point |
